# The Role of Current Density Distribution on Local Hardening of 20GL Steel During Electrolytic Plasma Processing

**DOI:** 10.3390/ma18225073

**Published:** 2025-11-07

**Authors:** Rinat Kurmangaliyev, Bauyrzhan Rakhadilov, Nurlat Kadyrbolat, Rinat Kussainov, Almasbek Maulit, Yeldos Mukhametov

**Affiliations:** 1 Engineering Center «Strengthening Technologies and Coatings», Shakarim University, Semey 071412, Kazakhstan; ersinnur44@gmail.com (N.K.); rinat.k.kus@mail.ru (R.K.); eldos_sports@mail.ru (Y.M.); 2 Department of Technical Physics and Heat Power Engineering, Research School of Physical and Chemical Sciences, Shakarim University, Semey 071412, Kazakhstan; tech_physics_energy@shakarim.kz; 3 Plasma Science LLP, Ust-Kamenogorsk 070000, Kazakhstan

**Keywords:** electrolytic plasma processing, current density, microhardness, discharge, hardening, COMSOL Multiphysics, corrosion rate

## Abstract

This study investigates the influence of current density distribution on the hardening behavior of 20GL cast steel during electrolytic plasma processing (EPP). Experimental and numerical methods were combined to establish the relationship between discharge dynamics, heat flux, microstructural transformation. Electrolytic plasma hardening was carried out at cathodic voltages of 150 V and 250 V in a 20% Na_2_CO_3_ solution. The transient evolution of current density was analyzed using a 3D COMSOL Multiphysics model incorporating a vapor–gas shell (VGS) represented as a distributed impedance layer with realistic conductivity and permittivity. High-speed video confirmed that microdischarges preferentially initiate at sample corners, where modeling also predicts local current concentration and heat flux up to 12 MW/m^2^. Experimental current density values (3–4 × 10^4^ A/m^2^) showed good agreement with the simulations. Microhardness tests revealed that increasing voltage from 150 V to 250 V increases the thickness of the hardened layer (from ~250 µm to ~600 µm) and raises surface hardness (up to 750 HV), while polarization tests showed a 40% reduction in corrosion rate. The results highlight that current density distribution governs the non-uniformity of thermal effects and surface strengthening during EPP, emphasizing the importance of electrode alignment and VGS stability for uniform hardening.

## 1. Introduction

One of the key ways to increase the wear resistance and durability of metal parts is through local hardening of the surface layers of steel. It is known that the mechanical properties and wear resistance of working surfaces largely determine the service life of products, leading to the development of various surface hardening methods, ranging from induction and laser processing to chemical–thermal saturation. However, traditional technologies often require significant energy costs, expensive equipment, and lengthy processing times [1]. In recent years, the technology of electrolytic plasma processing (EPP) of metals has been actively developed, enabling effective surface hardening at relatively low costs and in a short time. Electrolytic plasma heating and hardening of steel (also known as electrolytic plasma hardening, EPH) is a specialized heat treatment process where the combination of electrochemical heating and plasma discharge provides a highly concentrated local energy supply to the surface, followed by intensive cooling in the electrolyte [2].

EPP technology is distinguished by its versatility, availability, and environmental safety. It allows for not only hardening, but also polishing or chemical–thermal modification, depending on the processing modes. In order for electrolytic plasma to be generated between two solid electrodes in the electrolyte, the condition of inequality of the electrode areas S_1_ > 5S_2_ is necessary (S_1_ and S_2_ are the electrode areas). In this case, plasma will be generated on an electrode with smaller geometric dimensions [3].

The physical essence of the EPP method is as follows: the electrode (cathode) is immersed in a flowing aqueous electrolyte through which electric current passes between the cathode and the anode. Joule heating leads to the formation of a vapor–gas shell around the cathode surface, and when the critical voltage is reached, plasma microdischarges arise in the interelectrode gap. The resulting electrolyte plasma locally heats the surface layer above the austenitizing temperature (Ac_3_), followed by rapid quenching by the surrounding electrolyte, which serves as an effective cooling medium [4]. As a result, the surface layer transforms into a fine-grained martensitic structure (possibly with bainite), while the underlying metal retains its original ferritic–pearlitic microstructure [5].

In modern scientific literature (both domestic and foreign), special attention is paid to the influence of EPP parameters on the characteristics of the hardened steel layer. It is noted that the main properties of the modified surface—hardness, depth and microstructure of the hardened layer, as well as wear resistance—significantly depend on the process conditions, primarily on the current density and the distribution of this current over the surface, as well as on the applied voltage, the composition and temperature of the electrolyte and the heating time [6]. It is the spatial distribution of the current density during EPP that plays a key role, since it determines the local intensity of the thermal effect and the nature of the discharge occurring in different areas of the cathode surface. With uneven current density, plasma discharges are concentrated mainly in areas with increased current density, which leads to non-uniform temperature over the surface and, as a consequence, to a difference in the degree of hardening of different areas of the part. It has been established experimentally and by means of spectral methods that changes in the current flow modes affect the behavior of the electrolyte–plasma discharge: when the average density increases or the shape of the current changes, the intensity and number of microarc discharges change [7].

Current density distribution plays a key role in determining the nature of the discharges that arise and the intensity of the thermal effect. In [8], it is shown that during electrolytic-plasma treatment, the current distribution over the cathode surface is uneven, and in corner areas it can exceed the average value by more than an order of magnitude. This is due to the geometry features and the presence of local gradients of electric potential. Even a slight shift in the electrode from the center of symmetry leads to a redistribution of the current density by up to 50%, which in turn determines the heterogeneity of the formation of the vapor–gas shell and the local nature of the discharges.

In addition, the calculation of the potential and current density using models with a nonlinear volt–ampere characteristic and taking into account the resistance of the vapor–gas shell (VGS) confirms that the entire voltage load is concentrated on the VGS, and its resistance is the main factor determining the distribution of power and energy in the system. These results emphasize the need for numerical modeling of the geometry and topology of the current when designing EPP modes, especially for complex-shaped parts [8].

On the other hand, the studies of anodic EPP conducted in [9] show that with the anodic version of EPP, the current density is closely related not only to the electrical characteristics, but also to the electrochemical reactions at the phase boundary. With an increase in current density, due to a high concentration of electrolyte (for example, ammonium chloride), an increase in the thickness of the cemented layer is observed, due to increased diffusion and the “etching” of oxygen from the metal structure. The authors also indicate that the surface oxide layer and the products of electrode reactions play an important role in modulating local heat transfer and diffusion saturation of steel.

Thus, the current density and its local distribution act as a key control parameter in the formation of the structure and properties of the treated surface. This position is substantiated both experimentally and in numerical models covering both thermal and electrophysical aspects of the process. The development of effective technological solutions requires further study of the influence of the electrode shape, electrolyte composition and the flow of microarc discharges on the current distribution, which is the subject of this study.

The novelty of this work lies in a comprehensive investigation of the spatial–temporal distribution of current density during the electrolytic plasma treatment of 20GL steel, performed through a combination of experimental measurements, high-speed video diagnostics, and numerical modeling in COMSOL Multiphysics. For the first time, a vapor–gas shell (VGS) model based on a distributed impedance boundary condition was implemented for this system, enabling a quantitative assessment of the influence of the VGS impedance on local current concentration in the edge and corner zones of the cathodic surface.

In contrast to most previous studies, which were limited to steady-state electrostatic simulations, the present work employs a transient 3D model (up to 10 s) that captures the temporal evolution and redistribution of current density and reflects the system’s transition to a quasi-steady discharge regime. Experimental current–time characteristics were used to validate the numerical model and to establish correlations between the oscillatory behavior of discharges and the resulting hardening depth and uniformity of the treated layer.

## 2. Materials and Methods

To evaluate the influence of electrolytic plasma hardening modes on the mechanical properties of the workpieces, samples of grade 20GL as-cast steel measuring 25 × 20 × 15 mm^3^ were prepared and manually polished in a sequential manner using sandpaper with grain sizes ranging from P100 to P2500. Two processing modes were investigated: one with a cathode voltage of 150 V for 10 s, and another with a cathode voltage of 250 V for 10 s. The typical chemical composition of the 20GL steel used in this study is presented in Table 1.

**Figure 1:**
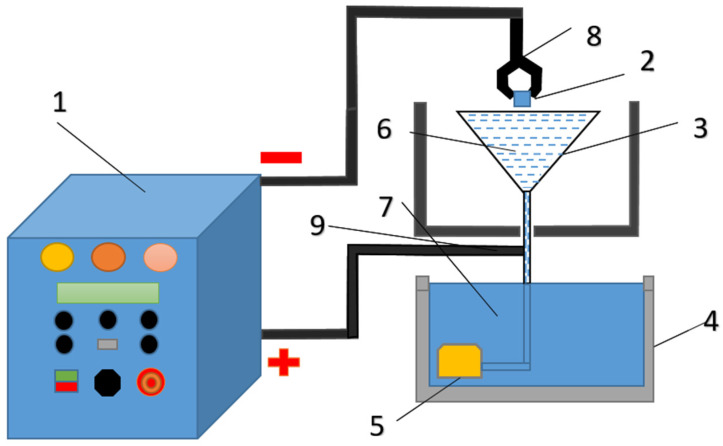
External appearance and basic diagram of the EPP installation: 1—power source; 2—workpiece (sample); 3—conical stainless steel electrolyzer; 4—sump; 5—pump; 6—electrolyte; 7—electrolyte bath; 8—cathode (−); 9—anode (+).

Electrolytic plasma hardening of grade 20GL steel samples and their subsequent study were conducted at the Engineering Center “Strengthening Technologies and Coatings” in Semey, Kazakhstan. A special installation designed for heating local areas of large-sized products was used to conduct EPH. The installation is a complex that includes a power source and an electrolytic cell, which are built into a chemical cabinet. A 50 kW power source provides a constant positive voltage of up to 380 V and a current of up to 150 A depending on the load. The power source is controlled by a digital module (Figure 1).

A 20% aqueous solution of sodium carbonate (Na_2_CO_3_) was used as the electrolyte. The electrolyte flow rate was maintained at approximately 5 L/min and was measured using an electronic flow meter Piusi K24 Meter (Piusi, Suzzara, Italy).

Video recording of the EPH process was carried out using a high-speed camera EVERCAM 2000-16-C (General Optics LLC, Moscow, Russia). Video recording was carried out at a frequency of 1000 frames per second. Two modes of cathodic electrolytic-plasma treatment were studied: at voltages of 150 and 250 V. The obtained video materials were processed in the SRV_HS program version 1.15 supplied by the camera manufacturer.

The microhardness of steel samples was measured using an HV-1 DT (Shanghai Hualong Test Instruments Corporation, Shanghai, China) device with an indenter load of *p* = 0.2 N and a holding time of 10 s (in accordance with the requirements of GOST 9450-76). The mean value was determined as the average of five indentation points located along a single line with a step of 10 µm.

For the comprehensive evaluation of the steel samples’ microstructure, a metallographic microscope HL-102AW (Shanghai Hualong Test Instruments Corporation, Shanghai, China) was employed. Sample preparation for metallographic microanalysis involved polishing sections with chromium dioxide paste, followed by etching with a 4% ethanolic solution of nitric acid.

The corrosion resistance tests of the 20GL steel samples were carried out at a temperature of +25 °C using the potentiodynamic polarization method on a CS300M potentiostat/galvanostat (Corrtest Instruments, Wuhan, China) in a 3% NaCl aqueous solution. A protective coating was applied to the sample surface, leaving an exposed area of 1 cm^2^, ensuring that all specimens had identical analysis areas and allowing consistent comparison of the corrosion behavior.

In the electrochemical cell (as part of the corrosion testing setup), the reference electrode was a silver/silver chloride (Ag/AgCl) electrode with a constant potential, the auxiliary electrode was a platinum plate, and the working electrode was the test sample made of 20GL steel. The cells were connected by a salt bridge, sealed on both sides with filter paper to prevent mixing of the NaCl-filled solutions. Prior to testing, all samples underwent surface preparation, including mechanical polishing, cleaning, and degreasing using ethanol.

Data acquisition and analysis were performed using CS Studio software version 6.3.1128.1 (Corrtest Instruments, Wuhan, China). The following electrochemical parameters were determined: corrosion potential (E_corr_), corrosion current density (j_corr_), corrosion rate. The obtained polarization curves were plotted in logarithmic form (log(j) vs. E), which enabled accurate evaluation of the electrochemical kinetics and comparison of corrosion resistance between the hardened and untreated samples.

To study the current density distribution during EPT, a three-dimensional numerical model was constructed using COMSOL Multiphysics 6.2 (COMSOL AB, Stockholm, Sweden). The Electric Currents (EC) module, which solves the current continuity equation coupled with Ohm’s law in integral form, was employed as the primary physics interface. The simulations were carried out in a time dependent formulation with a total simulation time of 10 s and a time step of 0.1 s, allowing the temporal evolution of the current density distribution and the establishment of a quasi-steady discharge regime to be observed.

The geometry consisted of a rectangular cathode (25 × 20 × 15 mm^3^) and a coaxially positioned conical anode with a base radius of 188 mm. The cathode was partially immersed in the electrolyte, while the upper section of the cone represented the anode surface. A constant negative potential was applied to the cathode surface (150 or 250 V), and a grounded potential (0 V) was set on the anode. The electrolyte domain between the electrodes was assigned electrical conductivity and relative permittivity values corresponding to the literature data [10,11].

A distinctive feature of the model is the inclusion of a thin impedance layer representing the vapor–gas shell at the cathode–electrolyte interface. The impedance boundary condition describes the interaction between the conductive surface and the surrounding medium by relating the tangential electric field to the induced surface current density. It effectively accounts for the finite conductivity and permittivity of the vapor–gas shell without explicitly modeling its entire volume. In the first approximation, this shell was modeled as an isotropic layer 100 μm thick [12], characterized by electrical conductivity and relative permittivity corresponding to a partially ionized water vapor–gas mixture. According to the literature [13,14,15,16], the specific conductivity of such mixtures typically lies in the range of 0.01–1 S/m, while the relative permittivity is ε_r_ ≈ 1.5–2.5. For the present study, averaged values of σ = 0.1 S/m and ε_r_ = 2.0 were adopted, ensuring a realistic distribution of potential and current lines in the cathode–electrolyte contact zone. This representation treats the VGS as a physical interfacial layer of finite thickness, possessing parameters characteristic of gas–plasma media.

**Figure 2:**
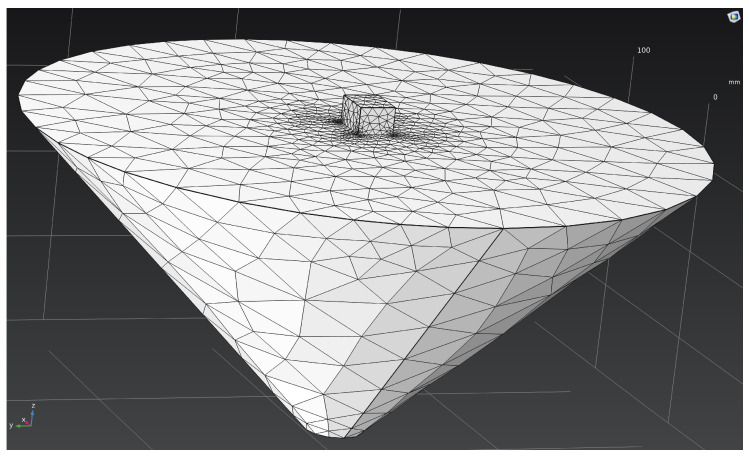
Finite element mesh of the computational model.

The computational domain was discretized using the finite element method (FEM) with a tetrahedral mesh and local refinement near the cathode surface, where the highest potential gradients and maximum current densities were observed (Figure 2). The mesh consisted of 7920 tetrahedral elements, 2008 triangular boundary faces, and 1771 nodes. The average element quality (evaluated using the skewness metric) was 0.68, with a minimum of 0.046, which corresponds to a good-quality mesh. The total volume of the computational domain was 6.81 × 10^6^ mm^3^.

The transient solution was obtained using automatic time-step control and adaptive convergence parameters. After the simulation, the spatiotemporal distribution of current density and the associated heat flux were analyzed, the latter being computed through the Electric Currents–Joule Heating multiphysics coupling.

## 3. Results and Discussion

**Figure 3:**
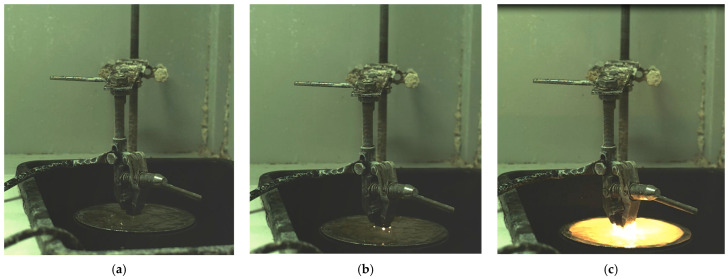
The moment of formation of a plasma discharge during the EPH process: (**a**) initial state of the system before discharge formation; (**b**) beginning of discharge breakdown at the corners of the cathode; (**c**) development of the discharge over the entire cathode surface.

Using a high-speed camera, images of the cathodic EPP process of a part made of 20GL steel were obtained (Figure 3a). It was found that the discharge began to form mainly at the sharp corners of the part (Figure 3b). After the discharge broke through at the corners, an avalanche-like discharge occurred (Figure 3c), after which the nature of the discharges repeated. The dynamics of the discharges were pulsed.

**Figure 4:**
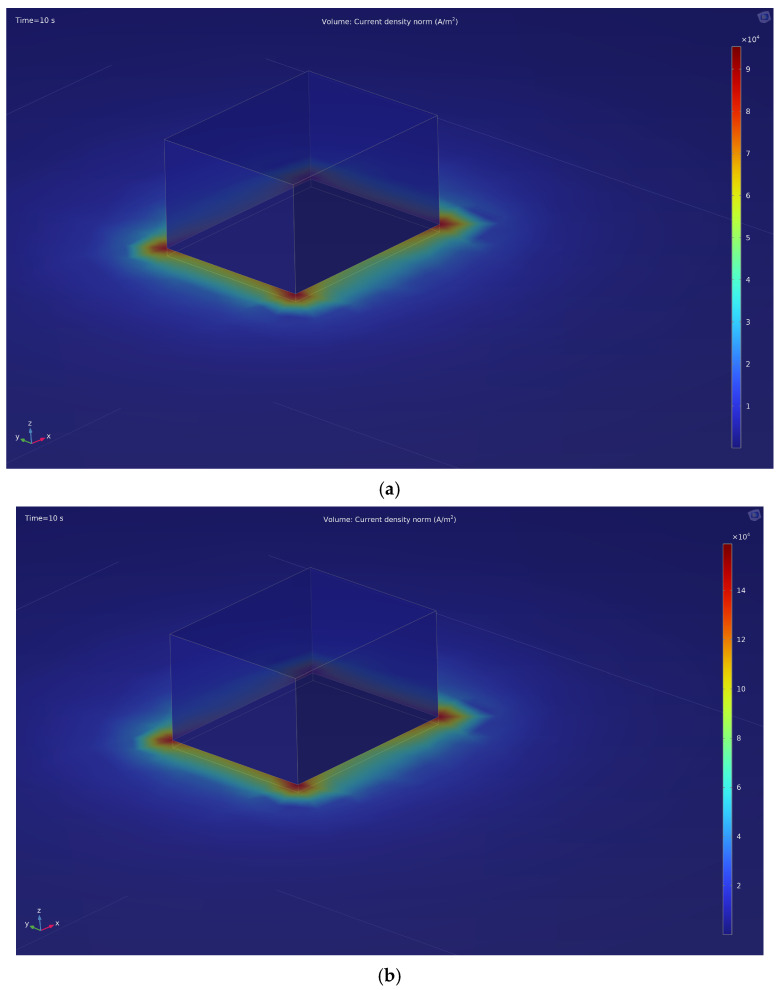
Distribution of current density at cathodic voltage: (**a**) 150 V; (**b**) 250 V.

This is also confirmed by computer modeling of the electric field during the EPP in the COMSOL Multiphysics program (Figure 4). The modeling also reveals that the current density near the sharp corners of the part has maximum values: at 150 V (Figure 4a), the maximum current density is 9 × 10^4^ A/m^2^, at 250 V (Figure 4b)—1.4 × 10^5^ A/m^2^.

**Figure 5:**
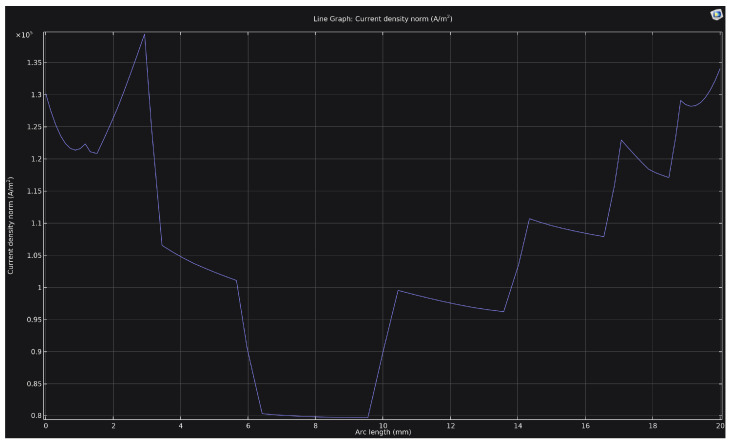
Current density distribution along a 20 mm-long rib at a voltage of 250 V (the end points correspond to the corners).

Figure 5 shows the distribution of current density along a 20 mm-long rib at a voltage of 250 V. It is evident that the density profile is non-uniform: the values decrease in the central region of the rib (down to approximately 0.8 × 10^5^ A/m^2^) and increase toward the corners, where the density reaches 1.3 × 10^5^–1.4 × 10^5^ A/m^2^. This distribution pattern results from the geometric enhancement of the electric field in the corner zones, leading to localized discharge concentration. Consequently, the corners and edges of the cathode are subjected to more intense heating and thermal effects, which may cause non-uniformity in the structure and properties of the hardened layer.

**Figure 6:**
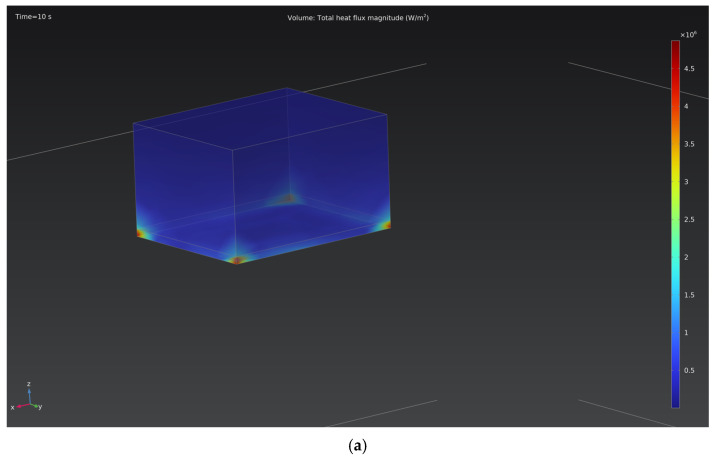

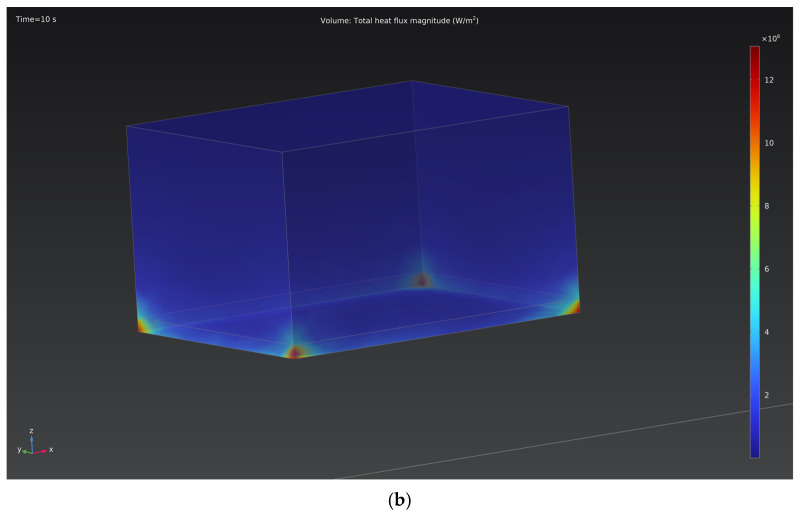
Distribution of heat flux for cathodic voltages of (**a**) 150 V and (**b**) 250 V.

Figure 6 shows the heat flux distribution patterns on the cathode surface for two processing modes: 150 V and 250 V. At the lower voltage, the maximum heat flux density does not exceed 4.5 MW/m^2^, whereas at 250 V, it increases to approximately 12 MW/m^2^, primarily near the edge regions. An increase in voltage leads to the intensification of local microarc discharges and a rise in the specific current density, which results in more pronounced thermal heating of the surface.

The obtained current density and heat flux values from the numerical simulation are in good agreement with the experimental data reported in the literature. In particular, it was observed in [17] that during cathodic plasma hardening of 14Kh17N2 steel with a treated area of about 16 cm^2^, the heat flux could reach up to 7.5 MW/m^2^, which is comparable to the present results at 250 V. It was shown in [18] that heat fluxes during anodic heating at 240 V reached 1.6–2.8 MW/m^2^, while the data in [19] indicate that typical current densities for plasma–electrolytic hardening of steels vary between 1.5 × 10^4^ A/m^2^ and 4.5 × 10^4^ A/m^2^. More recent study [20] have demonstrated that the current density during plasma–electrolytic treatment depends strongly on interelectrode spacing, ranging from 2 × 10^4^ to 5.8 × 10^4^ A/m^2^. The obtained numerical results also lie within the typical range of heat fluxes (5–15 MW/m^2^) reported in the literature [21], confirming the physical consistency of the modeling assumptions. Overall, these findings confirm that the obtained modeling results fall within a physically realistic range for EPP, adequately reflecting the heating behavior of the cathode region under typical treatment voltages.

**Figure 7:**
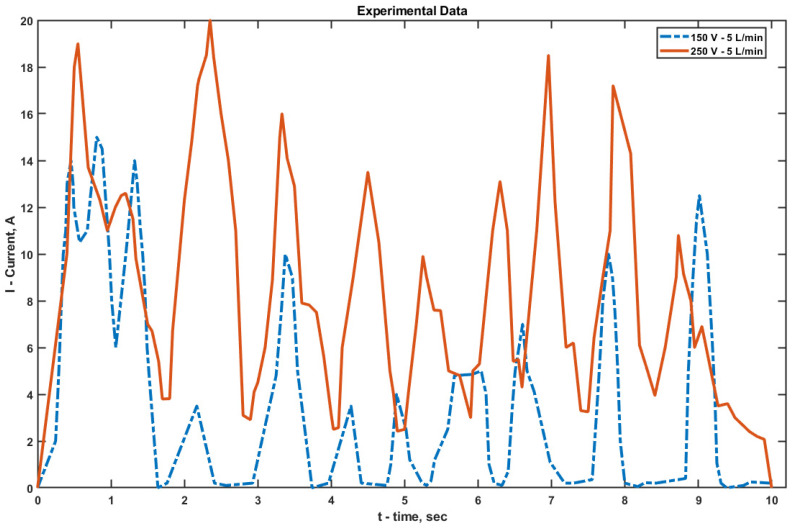
Dynamics of current strength in the system during 10 s of EPT at different voltages on the power source.

When analyzing the ammeter and voltmeter readings integrated into the power supply during EPP, it was found that each discharge corresponded to a distinct current peak. After each discharge, the current dropped sharply (almost to zero at 150 V), and the process repeated periodically (Figure 7). During cathodic EPT at 150 V, the maximum current reached 15 A, while at 250 V it increased to 20 A.

At 150 V, deep current drops down to zero were observed, which are associated with the complete rupture of the VGS and a relatively long recovery time under low voltage and average electrolyte flow (5 L/min). At 250 V, the pulse frequency remained approximately the same, but the discharge amplitude was significantly higher, and the VGS recovery period was slightly longer due to the denser structure of the vapor–gas shell and the increased temperature at the electrode–electrolyte interface.

Experimental estimates of the peak current density values (over an active area of approximately 5 cm^2^ and within the measurement time window) are on the order of 3 × 10^4^ A/m^2^ for 150 V and 4 × 10^4^ A/m^2^ for 250 V. The discrepancy with the simulated results (9 × 10^4^ A/m^2^ for 150 V and 1.4 × 10^5^ A/m^2^ for 250 V) is about 3–3.5 times, which is less than one order of magnitude. This deviation can be attributed to fundamental simplifications in the model: the vapor–gas shell (VGS) is represented as a stationary impedance layer without oscillatory dynamics, and both the geometry and electrodes are idealized—surface roughness, weld seams, micro-misalignment, and variations in immersion depth are neglected. Considering these assumptions, the obtained agreement can be regarded as satisfactory. In future work, it is planned to incorporate a time-dependent VGS impedance, as well as the coupling between shell thickness, temperature, and electric field, and to account for geometric tolerances, which should reduce the gap between simulation and experimental results.

**Figure 8:**
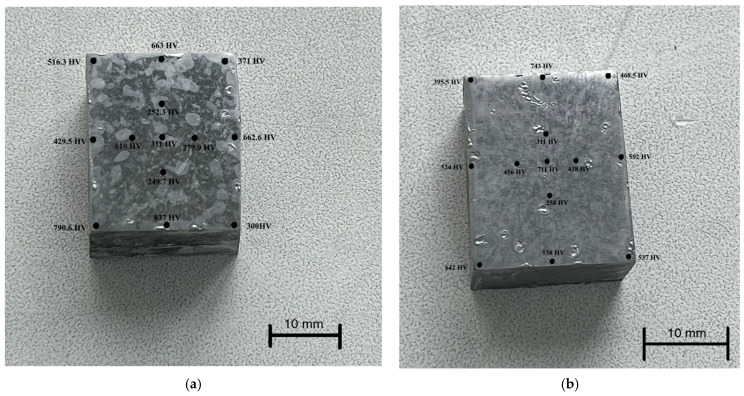
Distribution of microhardness over the surface of parts processed after EPH: (**a**) at 150 V; (**b**) at 250 V.

To determine the effect of the cathode mode of EPP on surface transformations in grade 20GL as-cast steel, the microhardness of the samples was determined. Figure 8 shows the distribution patterns of the Vickers microhardness over the surface of the parts. The initial hardness of the samples before EPP was about 150–160 HV.

From the analysis of Figure 8, a number of characteristic observations can be made. First, there is a stable tendency for hardness to increase at the edges of the samples. This confirms the assumption that it is the edge zones that are subject to the greatest localization of current, which is consistent with the results of numerical modeling of the current density distribution. Increased current density at the edges causes a more intense thermal effect and, as a consequence, an increase in the degree of hardening.

Secondly, despite the general pattern, there are pronounced fluctuations in the microhardness values, including local dips near the edge zones. Such heterogeneity can be caused by the instability of the electric discharge, a violation of the symmetry of the setup (for example, a displacement of the sample relative to the central axis of the electrolytic bath), and non-uniform formation of the vapor-gas shell on different parts of the surface. Similar effects were noted earlier in experimental studies, in particular in [8], where it was shown that a deviation in the position of a part even by 1–2 mm can significantly affect the current distribution and, as a consequence, the structure of the thermal effect.

This is especially evident when comparing samples processed at different voltages. At 250 V (Figure 8b), a more uniform increase in hardness is observed in the central zone of the sample compared to the 150 V mode (Figure 8a), which indicates the development of more powerful and possibly stable discharges with deeper thermal penetration. This may be due to exceeding the threshold for transition to the arc discharge mode, accompanied by intense local heating.

An additional factor contributing to local fluctuations of microhardness may be instabilities of the interphase boundary arising under conditions of electrolytic-plasma treatment. As shown in [22] and confirmed by a number of experimental studies [23], a significant contribution to the unevenness of heat and mass transfer is made by the implementation of the Tonks–Frenkel and Kelvin–Helmholtz instabilities. The first is associated with aperiodic deformations of the electrolyte surface charged due to a strong electric field, while the second is realized in the presence of a tangential shift between the vapor and liquid and is of an oscillatory nature. Their development leads to fluctuations in current density and temperature at the micro level, which, in turn, is reflected in the local kinetics of thermal diffusion processes and explains the observed unevenness of the hardness distribution even under macroscopically symmetric conditions [24,25,26]. Thus, taking into account electrohydrodynamic instabilities is an important element in interpreting the results of EPP and can form the basis for future models for predicting the properties of the modified layer.

**Figure 9:**
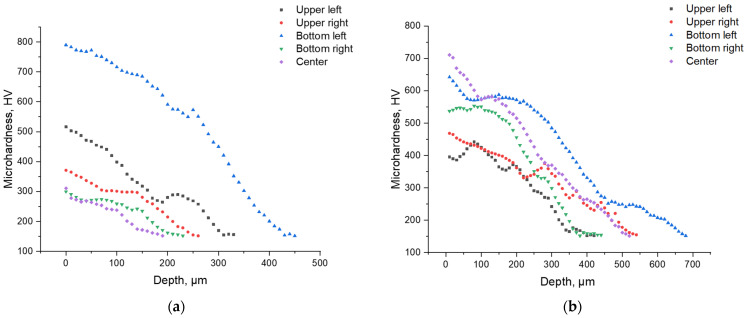
Microhardness distribution with depth: (**a**) 150 V; (**b**) 250 V.

To analyze the variation in hardness with depth, five strips were cut from each sample—four from the corners and one from the center. The Vickers microhardness was measured from the surface toward the core with a step of 20–30 µm. The resulting profiles are shown in Figure 9.

**Figure 10:**
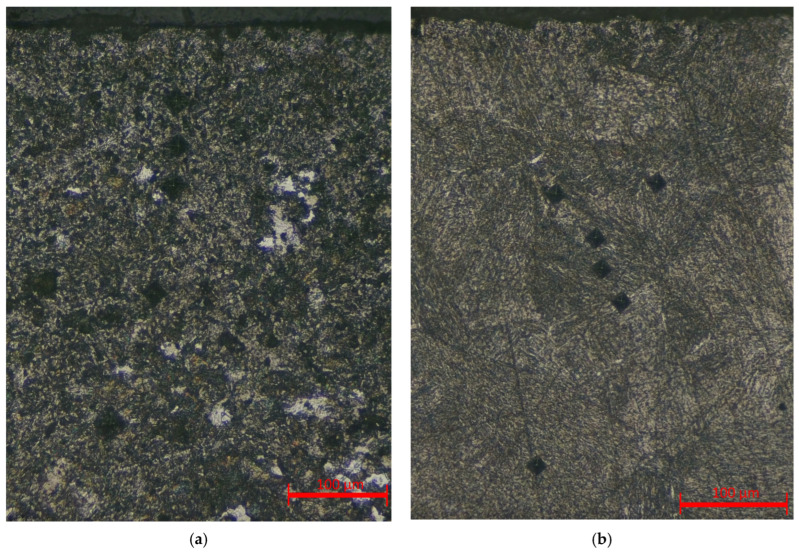
Optical micrographs (50×) of the central cross-sectional regions of 20GL steel samples after EPH at different voltages: (**a**) 150 V—hardened layer with a distinct transition to the ferritic–pearlitic core; (**b**) 250 V—near-surface region showing a fully martensitic structure (the ferritic–pearlitic core lies below the field of view).

As shown in the graphs, both modes exhibit a characteristic decrease in hardness from the surface toward the core, which is attributed to the temperature gradient and the gradual transformation from a martensitic to a ferritic–pearlitic structure. At 250 V, the maximum microhardness values reach 700–750 HV, and the thickness of the hardened layer extends to 500–600 µm, confirming a stronger thermal effect compared with the 150 V mode, where the surface hardness does not exceed 500 HV and the hardened zone is limited to 250–300 µm. The corresponding microstructural features of the hardened layers in the central cross-sections are shown in Figure 10.

Notably, in both cases, the highest microhardness values were recorded at the lower left edge of the samples, indicating localized intensification of heat flow in this region. This effect is likely caused by a slight displacement of the samples or by partial immersion of this edge deeper into the electrolyte, resulting in increased local current density and overheating. These findings highlight the importance of maintaining strict coaxial alignment of the electrodes and consistent sample positioning in the bath during EPP, particularly for components with complex geometry, to ensure uniform hardening across the surface.

The difference in current dynamics directly affects the thermal behavior of the system. At 150 V, the intermittent current flow and deeper interruptions limit heat penetration, leading to a shallower hardened layer. In contrast, at 250 V, the higher-amplitude pulses produce more intense thermal spikes, promoting the formation of a more stable and deeper hardened layer with enhanced uniformity.

**Figure 11:**
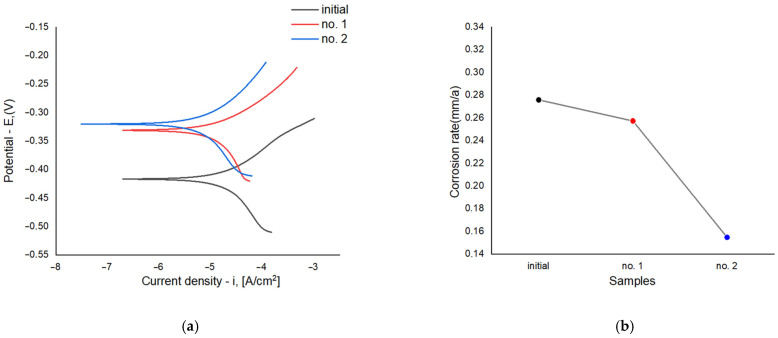
Potentiodynamic polarization curves (**a**) and corrosion rate (**b**) of 20GL steel before and after electrolytic-plasma hardening.

Figure 11a presents the polarization curves illustrating the relationship between current density and corrosion potential for samples before and after electrolytic plasma hardening (EPH)—the original sample, sample No. 1 treated at 150 V, and sample No. 2 treated at 250 V. Samples No. 1 and No. 2 exhibit a shift in corrosion potential toward more positive values and a reduction in corrosion current density compared with the original sample, indicating improved corrosion resistance.

Sample No. 1 shows a slightly lower corrosion current density (2.19 × 10^−2^ mA/cm^2^) than the original (2.35 × 10^−2^ mA/cm^2^), reflecting a moderate improvement in corrosion behavior. The best performance was observed for sample No. 2, which exhibited the lowest corrosion current density (1.32 × 10^−2^ mA/cm^2^), demonstrating the highest corrosion resistance after EPH.

Figure 11b shows the corrosion rate values for the three samples. The original sample exhibits the highest corrosion rate (0.2758 mm/year), indicating the lowest corrosion resistance among all specimens. After EPH, the corrosion rate decreases slightly for sample No. 1 (0.2573 mm/year). The lowest corrosion rate is observed for sample No. 2 (0.1549 mm/year), confirming its superior corrosion resistance.

## 4. Conclusions

This study experimentally and numerically confirmed that the spatial distribution of current density during electrolytic plasma processing (EPP) plays a decisive role in determining the efficiency and uniformity of surface hardening of 20GL steel. High-speed video recording revealed that microdischarges predominantly originate in regions with sharp geometric features, where local current concentration is highest. The numerical model reproduced this behavior, predicting a several-fold increase in current density and heat flux near the corners of the sample, with maximum values reaching approximately 9 × 10^4^ A/m^2^ and 4.5 MW/m^2^ at 150 V, and 1.4 × 10^5^ A/m^2^ and 12 MW/m^2^ at 250 V, respectively.

Analysis of the microhardness profiles demonstrated that the non-uniformity of current distribution leads to heterogeneous surface hardening, while increasing the voltage to 250 V promotes a more stable discharge regime and a deeper hardened layer. The detected non-uniformity in the distribution of microhardness may be due to a number of factors, such as the asymmetry of the sample position, variations in the formation of the vapor-gas shell, and electrohydrodynamic instabilities. All this can cause fluctuations in current and, accordingly, temperature, affecting the local structure of the processed layer.

Future research will focus on improving the physical accuracy of EPP modeling by incorporating the oscillatory dynamics of the vapor–gas shell (VGS) and its dependence on cathode temperature and electric field strength. It is also planned to analyze the contribution of Tonks–Frenkel and Kelvin–Helmholtz instabilities to the evolution of the interfacial plasma–liquid layer, which may significantly affect current fluctuations, heat transfer, and surface modification mechanisms.

Thus, the current density distribution in the system should be considered as a critically important parameter determining the efficiency of hardening and the homogeneity of the modified layer during EPP. The results obtained can be used in designing processing modes and electrode geometry to ensure the required characteristics of thermally modified zones.

## Figures and Tables

**Table 1:** Chemical composition of the 20GL steel.

C	Si	Mn	P	S	Fe
0.15–0.25	0.2–0.4	1.2–1.6	<0.4	<0.4	Bal.

## Data Availability

The original contributions presented in the study are included in this article. Further inquiries can be directed to the corresponding author.
